# Development and evaluation of luteolin loaded pegylated bilosome: optimization, *in vitro* characterization, and cytotoxicity study

**DOI:** 10.1080/10717544.2021.2008055

**Published:** 2021-12-06

**Authors:** Ameeduzzafar Zafar, Nabil K. Alruwaili, Syed Sarim Imam, Omar Awad Alsaidan, Mohd Yasir, Mohammed M. Ghoneim, Sultan Alshehri, Md. Khalid Anwer, Alanood S. Almurshedi, Abdullah S. Alanazi

**Affiliations:** a Department of Pharmaceutics, College of Pharmacy, Jouf University, Sakaka, Al-Jouf, Saudi Arabia; b Department of Pharmaceutics, College of Pharmacy, King Saud University, Riyadh, Saudi Arabia; c Department of Pharmacy, College of Health Sciences, Arsi University, Asella, Ethiopia; d Department of Pharmacy Practice, College of Pharmacy, AlMaarefa University, Ad Diriyah, Saudi Arabia; e Department of Pharmaceutics, College of Pharmacy, Prince Sattam Bin Abdulaziz University, Al-kharj, Saudi Arabia; f Department of Clinical Pharmacy, College of Pharmacy, Jouf University, Sakaka, Al-Jouf, Saudi Arabia; g Health Sciences Research Unit, Jouf University, Sakaka, Al-Jouf, Saudi Arabia

**Keywords:** Bilosomes, breast cancer, Box–Behnken’s design, luteolin, pegylated

## Abstract

The present research was aimed to develop luteolin (LL) loaded pegylated bilosomes (PG-BLs) for oral delivery. The luteolin bilosomes (BLs) were prepared by the thin-film hydration method and further optimized by the Box–Behnken design (four-factors at three-levels). The prepared LL-BLs were evaluated for vesicle size (VS), PDI, zeta potential (ZP), and entrapment efficiency to select the optimized formulation. The optimized formulation was further assessed for surface morphology, drug release, gut permeation, antioxidant, and antimicrobial study. The cytotoxicity study was conducted on breast cancer cell lines (MDA-MB-231 and MCF7). The optimized formulation LL-PG-BLs-opt exhibited a VS of 252.24 ± 3.54 nm, PDI of 0.24, ZP of −32 mV with an encapsulation efficiency of 75.05 ± 0.65%. TEM study revealed spherical shape vesicles without aggregation. The DSC and XRD results revealed that LL was encapsulated into a PG-BLs matrix. LL-PG-BLs-opt exhibited a biphasic release pattern as well as significantly high permeation (*p*<.05) was achieved vis-a-vis LL-BL-opt and LL dispersion. The antioxidant activity result revealed 70.31 ± 3.22%, 83.76 ± 2.56%, and 96.87 ± 2.11% from LL-dispersion, LL-BLs-opt, and LL-PG-BLs-opt, respectively. Furthermore, LL-PG-BLs-opt exhibited high cell viability on both cell lines than LL-BL-opt and pure LL. The IC_50_ value was found to be 390 µM and 510 µM against MCF7 and MDA-MB-231 cancer cells, respectively. The antimicrobial activity result exhibited LL-PG-BLs-opt had better antibacterial activity than pure LL against *Staphylococcus aureus* and *Escherichia coli*. Hence, PG-BLs might provide an efficient nano oral delivery for the management of the different diseases.

## Introduction

In breast cancer (BC), breast tissue is divided irregularly and forms a solid tumor. The symptoms of BC include swelling in the breast, change in shape, cellulitis of the skin, and discharge of fluid. BC has been considering one of the most common type of cancer worldwide. According to the World Health Organization (WHO), BC accounts for about 12% of all cancer cases annually. The most common elements for developing BC are lifestyle, breast condition, family history, age, and genetic mutation, etc. (Kamaruzman et al., 2019). However, postmenopausal women are more at risk of BC. About 7% of BC were reported in women of age 40 years and about <4% of age 35 years (Sharma et al., 2013). The key therapy for BC is surgery, radiotherapy, and chemotherapy. The conventional and biological therapies used for BC treatment are showing instability of the drug, toxicity to normal cells, and the chance of the development of the drug-resistant, failure to complete eradication of cancer cells and require high dose with long treatment schedule (Liang et al., 2010). Hence, the formulation of new chemotherapeutic agents presents significant importance for the management of BC.

In the last two decades, many herbal compounds were used for antibacterial, antioxidant, and anticancer activities. Many flavonoids, alkaloids, and terpenes act as anticancer and antibacterials by increasing the membrane permeability and preventing enzyme synthesis (Mencherini et al., 2007). Luteolin (LL) is a bioactive flavonoid found in various plants and vegetables (Lin et al., 2008). Chemically, it is a 3,4,5,7-tetra hydroxy flavone having the molecular weight and log *p* value as 286.24 and 2.26 g/mol, respectively. It is soluble in organic solvent and sparingly soluble in water (1.93 × 10^−5^ mol/kg at 20 °C) (Eichsteininger et al., 2019; Alshehri et al., 2020). There are various pharmacological activities reported for LL such as anticancer, antioxidant, antibacterial, and anti-inflammatory (Boeing et al., 2020; Guo et al., 2020). It kills the various gram-positive and gram-negative bacteria like *Pseudomonas fluorescens*, *Listeria monocytogenes*, *Bacillus subtilis*, *Staphylococcus aureus*, and *Escherichia coli.* It acts by preventing nucleic acid and protein synthesis as well as disrupts the cell membrane (Qian et al., 2020). LL exhibited an anti-cancer effect in the different solid tumors by the initiation of apoptosis, stopping the cell cycle as well as by impeding the cell proliferation (Imran et al., 2019). The poor soluble property strictly limits the applications in pharmaceutical product development. Several research studies have been reported for different LL formulations like chitosan-coated NLCs (Gilani et al., 2021), nano emulsifying system (Ansari et al., 2020), solid self nano emulsifying system (Zhang et al., 2020), zein nanoparticle (Shinde et al., 2019), liposome (Wu et al., 2018), phospholipid complex (Khan et al., 2016), etc. The lipid-based nanocarrier systems have been used to enhance the solubility as well as therapeutic activity. These systems include eplerenone nanostructured lipid carriers (NLCs) (Abd-Elhakeem et al., 2021), rosuvastatin liposomes (Ahmed, 2020), lovastatin hybrid liposome (Romana et al., 2020), glycyrrhizic acid and cisplatin nanoliposomes (Hatami et al., 2020), *Fumaria officinalis* niosomes (Raafat & El-Zahaby, 2020), apigenin, piceatannol bilosomes (Alhakamy et al., 2021; Zafar et al., 2021), and olmesartan medoxomil pegylated bilosomes (PG-BLs) (Albash et al., 2019). Among them, PG-BLs are the new approach for the improvement of the therapeutic efficacy of the poorly soluble drugs. PG-BLs consist of lipid, surfactant, bile salt, and polyethylene glycol (PEG). PG-BLs are similar to liposomes but it protects the drug from enzymatic degradation in the GIT system. It can overcome the problem associated with liposomes like encapsulation, stability, lysis of vesicle, and scaling up the problem (Albash et al., 2019). Bile salts (sodium deoxycholate, SDC) are a nontoxic, biocompatible, solubilizing agent, and also reported as permeation enhancer (Deng & Bae, 2020). The PG-BLs offer high drug loading and modulate the drug release over an extended period. PG decreases the affinity to eradicate the formulation through the reticuloendothelial system, enhances the mucoadhesion as well as improves the therapeutic efficacy by enhancing circulation time (Nag & Awasthi, 2013; Dai et al., 2016; Suk et al., 2016).

In this research work, we formulated LL-loaded pegylated bilosomes **(**LL-PG-BLs) and optimized them by the Box–Behnken design using different independent variables (Table 1). The formulations were characterized for physicochemical parameters, permeation study, cell viability study, antioxidant study, and antimicrobial study.

**Table 1.** Optimization variables and their responses used for Box–Behnken’s design software.

Factor	Levels used		
	Low level (–1)	Middle level (0)	High level (+1)
*Formulation variables*			
* A*: Surfactant (Span 60, mg)	100	200	300
* B*: Cholesterol (CHOR, %)	1	3	5
* C*: Bile salt (mg)	0.15	0.25	0.35
* D*: PEG-2000 (mg)	1	2.5	4

Responses	Constrains
*Y*_1_: vesicle size (VS)	Minimized
*Y*_2_: entrapment efficiency (EE)	Maximized

## Requirements and experimental

### Requirements

Luteolin was procured from the Beijing Mesochem Technology Pvt. Ltd. (Beijing, China). Cholesterol and Span 60 were procured from SD Fine Ltd. (Mumbai, India). Sodium deoxycholate and polyethylene glycol 2000 (PEG-2000) were purchased from Lobachem (Colaba, Mumbai, India). Chloroform, methanol, and dialysis membrane (MWCO, 12,000 kDa) were obtained from Sigma-Aldrich (St. Louis, MO). Other chemicals used for the study were of analytical grade.

### Experimental

#### Development of luteolin-loaded pegylated bilosomes

The thin-film hydration method was utilized for the preparation of LL-PG-BLs using cholesterol, span 60, bile salt, PEG, and LL (Albash et al., 2019). The ingredients were dissolved in organic solvent (12 mL, chloroform: methanol, 1:1). The mixture was filled into round bottom flask and fixed to the rotary evaporator (IKA, RV-3V-C, Staufen, Germany) at 40 °C (water bath) with 100 rpm. The organic solvent was evaporated at reduced pressure and the thin film was formed to the wall of flask. The flask was kept in desiccator for 24 h to remove the remaining solvent. Then, the thin film was hydrated for 45 min with 10 mL of Milli-Q water. Then, LL-PG-BLs dispersion was sonicated for 5 min in a water bath sonicator (Branson CPX-952-518R, Branson, Danbury, CT). The conventional bilosomes was prepared by using span 60 (45 mg), CHOR (25 mg), and SDC (12.5 mg) by similar method for comparison study.

#### Box–Behnken design optimization

The optimization of LL-PG-BLs was done by using four-factors at three-levels Box–Behnken design (version 10, Stat-Ease Inc, Minneapolis, MN). The surfactant (*A*), cholesterol (CHOR, *B*), bile salt (BL, sodium deoxy cholate, *C*) PEG 2000, (*D*) were used as independent variables **(**Table 1). Their influence was determined on vesicle size (VS) (*Y*_1_) and entrapment efficiency (EE) (*Y*_2_). The design showed 27 formulation runs with five center points (Table 2). The results of both responses were fitted into various experimental design models (linear, 2nd order, and quadratic model) to determine the best fit model on basis of statistical analysis (*R*^2^). The fitted model was further evaluated by analysis of variance (ANOVA). The mathematical polynomial equation and 3D-surface plot were constructed for the analysis of formulation variable over the responses.

**Table 2.** Formation composition of apigenin bilosomes (APG-BLs) with actual and predicted value of the vesicle size (*Y*_1_) and entrapment efficiency (*Y*_2_).

Code	*A* (mg)	*B* (mg)	*C* (mg)	*D* (mg)	*Y*_1_ (nm)		*Y*_2_ (%)	
					Actual	Predicted	Actual	Predicted
LL-PG-BLs 1	30	15	12.5	30	235	238	53	51
LL-PG-BLs 2	60	15	12.5	30	100	101	68	66
LL-PG-BLs 3	30	35	12.5	30	283	280	76	75
LL-PG-BLs 4	60	35	12.5	30	285	284	88	87
LL-PG-BLs 5	45	25	5	20	248	247	69	68
LL-PG-BLs 6	45	25	20	20	266	265	77	74
LL-PG-BLs 7	45	25	5	40	324	326	67	64
LL-PG-BLs 8	45	25	20	40	243	245	76	75
LL-PG-BLs 9	30	25	12.5	20	263	262	65	64
LL-PG-BLs 10	60	25	12.5	20	200	202	80	83
LL-PG-BLs 11	30	25	12.5	40	300	298	66	64
LL-PG-BLs 12	60	25	12.5	40	223	225	75	73
LL-PG-BLs 13	45	15	5	30	205	203	58	56
LL-PG-BLs 14	45	35	5	30	325	327	78	79
LL-PG-BLs 15	45	15	20	30	185	183	66	64
LL-PG-BLs 16	45	35	20	30	281	284	86	88
LL-PG-BLs 17	30	25	5	30	296	295	60	59
LL-PG-BLs 18	60	25	5	30	236	233	76	75
LL-PG-BLs 19	30	25	20	30	268	267	70	72
LL-PG-BLs 20	60	25	20	30	200	197	80	78
LL-PG-BLs 21	45	15	12.5	20	179	179	63	61
LL-PG-BLs 22	45	35	12.5	20	290	288	83	83
LL-PG-BLs 23	45	15	12.5	40	206	205	60	58
LL-PG-BLs 24	45	35	12.5	40	320	321	83	82
LL-PG-BLs *25	45	25	12.5	30	260	256	72	74
LL-PG-BLs *26	45	25	12.5	30	254	256	71	74
LL-PG-BLs *27	45	25	12.5	30	256	256	75	74

*A*: surfactant (mg); *B*: cholesterol (%); *C*: bile salt (mg); *D*: PEG-2000 (mg); *Y*_1_: vesicle size (nm); *Y*_2_: entrapment efficiency (%).

### Characterization

#### Vesicle characterization

The VS, polydispersity index (PDI), and zeta potential (ZP) of the prepared formulations were measured by zeta sizer (HAS 3000, Malvern Instruments, Malvern, UK). The diluted LL-PG-BLs sample was filled into a disposable cuvette and evaluated for the size, PDI, and ZP.

#### DSC study

The DSC analysis of LL, BS, PEG2000, CHOR, and LL-PG-BLs-opt was done by using the DSC instrument (Mettler-Toledo, Columbus, OH). Each sample (∼3 mg) was individually packed into an aluminum pan and placed into the instrument for the scanning between 25 and 400 °C under nitrogen gas (inert condition provider).

#### X-ray diffraction study

XRD analysis of LL, PEG2000, CHOR, and LL-PG-BLs-opt was done by using XRD instrument (Diffractometer Ultima IV, Rigaku, Woodland, TX). The samples were filled to the sample holder and placed into the instrument. The sample was scanned at a 2 theta level between 5 and 60°, operated at 40 kV voltage, 30 mA current (copper tube), with scanning speed of 2.0°/min.

#### In vitro release study

The release study was performed by using a dialysis membrane to compare the release pattern (Parashar et al., 2019). The samples were filled into the pretreated dialysis bag and tied from both ends. The bags were fixed with a stand and placed in a beaker containing 250 mL phosphate buffer saline (pH 7.4). The study was performed at a stirring speed of 100 rpm and temperature was maintained for 37 ± 0.5 °C. The released content of each sample (LL-dispersion, LL-PG-BLs-opt, and LL-BLs-opt) was collected at a definite time and the same volume of fresh medium was added to maintain the sink condition (concentration gradient). The samples were diluted and LL concentration in each sample was determined using a UV-visible spectrophotometer (Shimadzu-1800, Kyoto, Japan) at 340 nm. The study was conducted in triplicate and the graph was plotted between cumulative % drug release vs. time. The release data of LL-PG-BLs-opt formulation were fitted into various kinetic models, i.e. zero-order (*F*=k_0_t), first-order (Log *Q_t_*=log*Q*_o_ – *k*_1_*t*/2.303, *Q_t_*=amount of drug release in time *t*, *Q*_o_=initial amount of drug con), Higuchi (*M*_0_ – *M_t_*=*kt*^1/2^), Korsmeyer-Peppas (Log(*M*_0_ – *M_t_*)=log*k*+*n* log *t*), and Hixson-Crowell model (*Q*_o_^1/3^ – *Q*_t_^1/3^=*K*_HC_*t*) to find out the best fit model.

#### Ex vivo permeation study

The *ex-vivo* intestinal permeation study of LL-dispersion, LL-BLs-opt, and LL-PG-BLs-opt was performed using the excised rat intestine. The fresh intestine was collected from the rat and washed with Tyrode’s solution to perform the study. Each formulation (∼5 mg of LL) was filled into the intestine and tightly tied from both ends. The intestine was immersed in Tyrode’s solution (100 mL) as a release medium. During the study, the sample was aerated with 95% O_2_, and the temperature was maintained at 37 ± 0.5 °C with stirring speed of 100 rpm. At a predetermined time (30, 60, 90, and 240 min), permeated content (2 mL) was withdrawn and simultaneously replaced with the same volume. LL concentration was determined by the previously reported HPLC method (Zhang et al., 2007). The flux was calculated and the apparent permeability coefficient (APC) was determined by the following equation:

(1) $$\mathrm{APC}=\frac{\mathrm{flux}}{\text{initial concentation }\times \text{surface area }}\;$$

#### In vitro antioxidant activity

The anti-oxidant activity of pure LL, LL-BLs-opt, and LL-PG-BLs-opt dispersion was done by the previously reported method with slight variation (Zarai et al., 2013). The stock solution (1 mg/mL) of pure LL, LL-BLs-opt, and LL-PG-BLs-opt was prepared in organic solvent and further diluted in the concentration range of 25–500 µg/mL. The ethanolic solution (0.02% v/v) of 2,2-diphenyl-1-picrylhydrazyl (DPPH) solution (500 µL) was added to each sample and vortexed. The samples were kept in dark for 1 h at room temperature complete the reaction. The antioxidant molecules have the property to change the color. The analysis was carried out by a UV-visible spectrophotometer (Shimadzu 1800, Kyoto, Japan) at 517 nm. The antioxidant activity relative to control was calculated by the given formula:

(2) $$\%\;\mathrm{Radical}\;\mathrm{scavenging}\;=\frac{\mathrm{Abs}\;\mathrm{of}\;\mathrm{control}-\mathrm{Abs}\;\mathrm{of}\;\mathrm{the}\;\mathrm{treated}\;\mathrm{sample}}{\mathrm{Abs}\;\mathrm{of}\;\mathrm{control}}\times 100$$

### Cell culture and cytotoxicity study

Cytotoxicity study of the formulation was carried out on BC cell line, i.e. MDA-MB-231 and MCF7 as per previously reported procedure with slight modification (Yang et al., 2007; Ayob et al., 2014). The cells were grown into Dulbecco’s modified Eagle’s medium (DMEM) containing penicillin (100 U/mL), streptomycin (100 µg/mL), and fetal bovine serum (FBS, 10%w/v) as a supplement for proper growth of the cell. The cell was incubated into CO_2_ humidified incubator (5% CO_2_) at 37 ± 0.5 °C. The test was performed with 3-(4,5-dimethylthiazol-2-yl)-2,5-diphenyltetrazolium bromide (MTT, Sigma, St. Louis, MO). The 100 µL cell line suspension (5 × 10^4^ cells/mL) was filled into 96-well plate and incubated for 24 h. The different concentrations of various formulations like pure LL, LL-BLs-opt, and LL-PG-BLs-opt (25, 50, 100, 250, 500, and 1000 µM for MCF7 and 50, 100, 500, 1000, and 1500 µM for MDA-MB-231) were prepared in DMSO. Each sample (500 µL) was added to the microplate and incubated for 24 h and 48 h. After that 20 µL (5 mg/mL in phosphate buffer saline) was added and further incubated for 4 h at 37 °C. Then, formazan crystals were solubilized by adding 100 µL of DMSO into the micro-plate for completion. The absorbance was determined by the microplate reader (BioRad, Shinagawa-Ku, Japan) at 575 nm to calculate IC_50_ and % cell viability:

(3) $$\text{\% Cell viability}=\;\frac{\text{OD control}-\text{OD treated}}{\text{OD control}}\;\times 100$$

### Antimicrobial activity

The antibacterial activity of LL, LL-PG-BLs-opt, and pure ciprofloxacin solution were evaluated on *Staphylococcus aureus* (*S. aureus*, RCMB-010010) and *Escherichia coli* (*E. coli*, RCMB-010052) by the cup plate method (Adamczak et al., 2019; Guo et al., 2020). The petri plates were prepared by pouring of bacterial suspension and sterilized melted agar media and stand for solidification. The wells were made by using of sterile borer. The pure LL, LL-PG-BLs-opt dispersion (250 µg/mL), and pure ciprofloxacin solution were added into the well and stand for 15 min. The plates were placed into an incubator (Binder, Bohemia, NY) at 37 ± 0.5 °C for 24 h. The zone of inhibition was measured using graduated scale.

### Stability study

Stability study of LL-PG-BLs-opt was done at two different temperatures (4 °C and 25 °C/60% RH). The LL-PG-BLs-opt was sealed in borosilicate glass vials and placed into stability chamber (Thermo Fisher Scientific, Waltham, MA) for three months. At the definite time (0, 30, 60, 90, and 180 days), the sample was withdrawn and analyzed for the VS and EE.

## Results and discussion

### Optimization

**Figure 1.**
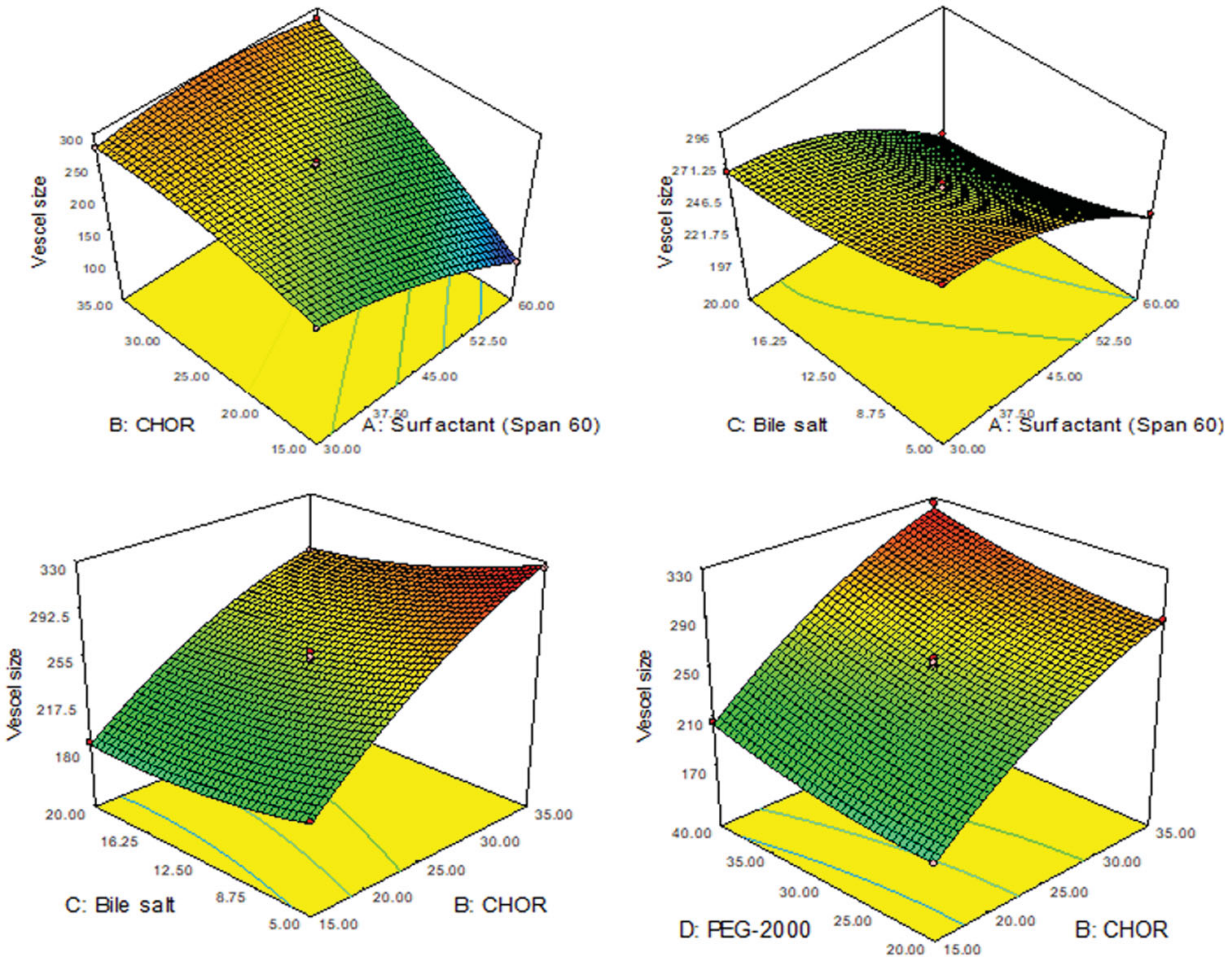
3D plots showing the influence of formulation variables. (A) Surfactant (Span-60, mg), (B) cholesterol (CHOR, %), (C) bile salt (mg), and (D) polyethylene glycol-2000 (mg) on the vesicle size.

**Figure 2.**
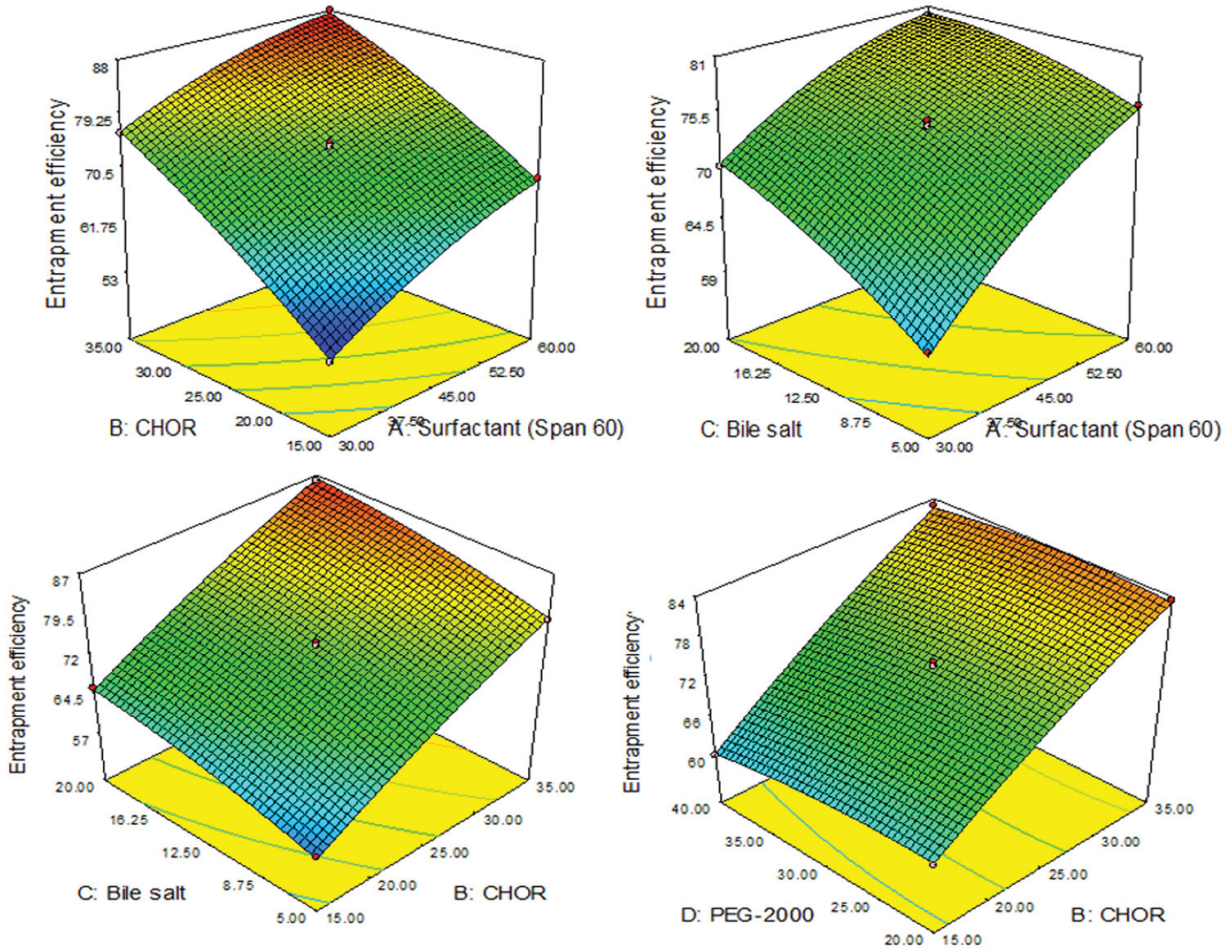
3D plots showing the influence of formulation variables. (A) Surfactant (Span 60, mg), (B) cholesterol (CHOR, %), (C) bile salt (mg), and (D) polyethylene glycol-2000 (mg) on the entrapment efficiency (%).

**Figure 3.**
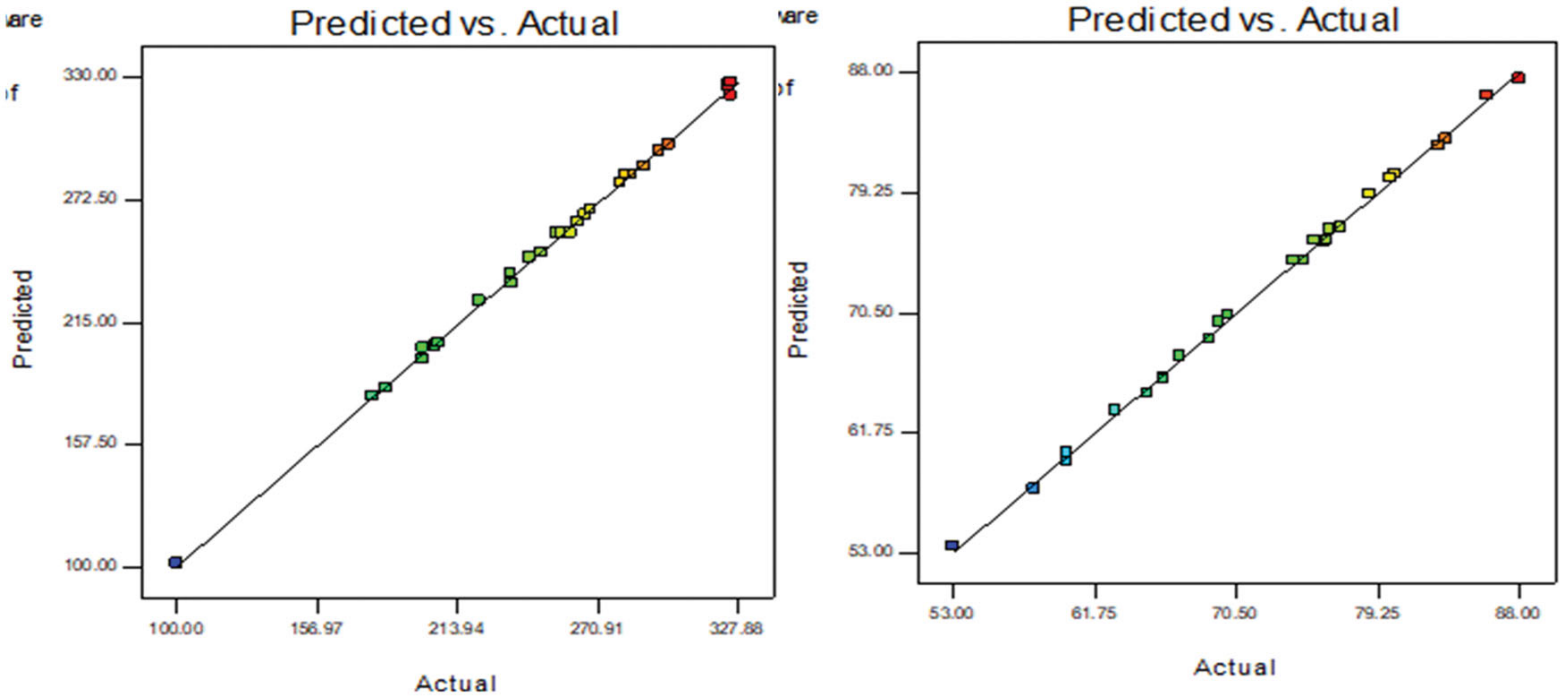
Actual and predicted plots of LL-PG-BLs showing the effect on vesicle size (nm) and entrapment efficiency (%).

LL-PG-BLs were developed by the thin-film hydration method and optimized by Box–Behnken’s design software. The design showed 27 formulations (LL-PG-BLs-1 to LL-PG-BLs-27) and the result of actual and predicted value of VS (*Y*_1_) and EE (*Y*_2_) are given in Table 2. The actual value of VS (*Y*_1_) and EE (*Y*_2_) were found to be in the range 100–325 nm and 53–88%, respectively. The actual values were found very close to the predicted values. The results were fitted to various statistical models and suggested quadratic model as the best fit model. For both responses, the *R*^2^ value of the quadratic model was found to be the highest (0.9985 for *Y*_1_ and 0.9984 for *Y*_2_) among all models (linear, 2nd order) (Table 3). Similarly, the model *F*-value for both responses was also highest (575.39 for *Y*_1_ and 568.11 for *Y*_2_) than other models. The model was further evaluated by ANOVA for both the responses and results were expressed in Table 4. The ANOVA value showed the effect of formulation factors (*A*, *B*, *C*, *D*) on responses individually, their interactions (*AB*, *AC*, *AD*, *BC*, *BD*, *CD*), and quadratic effects (*A*^2^, *B*^2^, *C*^2^, *D*^2^). The comparison was made at *p*<.05 for significant effect. 3D-surface graph of responses *Y*_1_ and *Y*_2_ explained the individual and interaction effect of variables on these responses (Figures 1 and 2). The actual and predicted values of each variable are shown graphically in Figure 3.

**Table 3.** Model statistical summary of all applied models for vesicle size and entrapment efficiency.

Source	Std. Dev.	*R* ^2^	Adjusted *R*^2^	Predicted *R*^2^	PRESS	Remark
*Vesicle size (Y_1_)*						
Linear	23.32	0.8259	0.7943	0.72679	18786.54	
2FI	16.53	0.9363	0.8966	0.79256	14264.05	
Quadratic	2.92	0.9985	0.9968	0.99239	523.6022	Suggested
*Entrapment efficiency (Y_2_)*						
Linear	1.46	0.9776	0.9734	0.9679	66.70	
2FI	1.17	0.9895	0.9830	0.9800	41.53	
Quadratic	0.51	0.9984	0.9967	0.9916	17.34	Suggested

**Table 4.** ANOVA evaluation of best fitted quadratic model for vesicle size and entrapment efficiency.

Source	Vesicle size (*Y*_1_)				Entrapment efficiency (*Y*_2_)			
	Sum of squares	df	*F* Value	*p* Value Prob>*F*	Sum of squares	df	*F* Value	*p* Value Prob>*F*
Model	68658.83	14	575.39	<.0001	2076.57	14	568.11	<.0001
*A*: surfactant (Span 60)	13240.37	1	1553.45	<.0001	511.97	1	1960.92	<.0001
*B*: CHOR	37985.85	1	4456.76	<.0001	1337.37	1	5122.34	<.0001
*C*: bile salt	3005.04	1	352.57	<.0001	173.72	1	665.39	<.0001
*D*: PEG-2000	2562.84	1	300.69	<.0001	9.97	1	38.21	<.0001
*AB*	4944.13	1	580.08	<.0001	4.23	1	16.18	.0017
*AC*	16	1	1.87	.1957	9.42	1	36.07	<.0001
*AD*	48.81	1	5.73	.0339	9.02	1	34.54	<.0001
*BC*	135.21	1	15.86	.0018	0.14	1	0.54	.4751
*BD*	16.12	1	1.89	.1941	1.58	1	6.05	.0300
*CD*	2432.46	1	285.39	<.0001	0.52	1	2.01	.1814
*A* ^2^	1382.57	1	162.21	<.0001	16.2	1	62.13	<.0001
*B* ^2^	1130.33	1	132.61	<.0001	6.07	1	23.26	.0004
*C* ^2^	330.39	1	38.76	<.0001	6.08	1	23.31	.0004
*D* ^2^	236.08	1	27.69	.0002	4.39	1	16.85	.0015
Residual	102.27	12	–	–	3.13	12	–	–
Lack of fit	83.61	10	0.8958	.6349	2.93	10	2.95	.2791
Pure error	18.66	2	–	–	0.19	2	–	–
Cor total	68761.11	26	–	–	2079.70	26	–	–

#### Effect of independent variables over vesicle size (Y_1_)

The polynomial equation for expressing the effect of the independent variable over VS is given below:

(4) Vesicle size (Y1)=+256.67−33.22 A+56.26 B−15.82C+14.61 D+35.16 AB− 2.00 AC−3.49 A D−5.81 BC+2.01 B D−24.66 CD−16.10 A2−14.56 B2+7.87 C2+6.65 D2

The positive and negative signs represented the synergistic and antagonistic effect of independent variables on the VS. Among all independent variables, the surfactant (span-60, A) and bile salt (C) showed negative effect. Whereas CHOR (B) and PEG-2000 (D) displayed positive effect on VS. The prepared LL-PG-BLs was found in the range of 100 nm (LL-PG-BLs-2) to 325 nm (LL-PG-BLs-14). There was significant variation in the size was observed. On increasing the concentration of surfactant (span-60), VS was decreased **(**Figure 1, Table 2) due to the presence of surfactant in BLs which help to reduce the interfacial tension between lipid and aqueous phase. This result agreed with previously published research work, i.e. topical bilosome delivery of tizanidine and tenoxicam (Al-Mahallawi et al., 2015; Khalil et al., 2019). Moreover, the increase in CHOR (B) concentration the VS also increases due to the increased hydrophobicity which leads to disorder in vesicle membrane (Patel et al., 2012). In addition, on increasing the bile salt, VS was significantly (*p*<.0001) decreased (Figure 1). This might be due to increasing the flexibility of BLs as well as decreased interfacial tension (Chen et al., 2009; Guan et al., 2011; Aburahma, 2016). The bile salt also participated in surface charge in vesicles. It provides a negative charge on the surface vesicle (Albash et al., 2019). The fourth factor, i.e. PEG-2000 was also significantly affected the VS but its effect was less prominent than other factors. On increasing PEG-2000 concentration, the VS increased due to the formation of a thin layer around the vesicles. From the ANOVA result, the model terms *A*, *B*, *C*, *D*, *AB*, *AD*, *BC*, *CD*, *A*^2^, *B*^2^, *C*^2^, *D*^2^ exhibited significant (*p*<.05) impact on VS, and the remaining terms exhibited a non-significant (*p*>.5) impact on VS (Table 4). The model *F*-value was found to be 575.39 (*p*<.0001) implied the model was well fitted. The lack of fit was non-significant (*p*>.05). The precision <4 (104.09) indicates an adequate signal for the fitted model. The predicted *R*^2^ (0.9924) was found to be in reasonable agreement with the adjusted *R*^2^ (0.9968) revealed that the quadratic model was well fitted.

#### Effect of independent variables over entrapment efficiency (Y_2_)

The polynomial equation for expressing the effect of the independent variable over EE is given below:

(5) Entrapment efficiency (Y2)=+74.34+6.53A+10.56B+3.80C−0.91D−1.03AB−1.53AC−1.50AD−0.19BC+0.63BD+0.36CD−1.74 A2−1.07B2−1.07C2−0.91D2

The positive and negative signs represent the synergistic and antagonistic effect of independent variables over the EE (*Y*_2_). From Equation (5), surfactant (span-60, *A*), CHOR (*B*), and bile salt (*C*) exhibited positive and PEG-2000 (*D*) showed a negative effect on the EE (Figure 2, Table 2). The EE (%) of LL-PG-BLs was found in a range of 53 (LL-PG-BLs-1) to 88% (LL-PG-BLs-4). On increasing the concentration of surfactant (span-60), the EE of LL increased because it stabilizes the lipid membrane. The increase in surface area of vesicle due to reduction of interfacial tension as well as due to low HLB value (4.7) of span 60 (AbouSamra & Salama, 2017). The second factor, i.e. CHOR (*B*) concentration increases the EE (%). It might be due to increased membrane rigidity (Patel et al., 2012). Moreover, it also enhances the firmness of the lipid bilayer membrane, permeability, stability and prevents the leakage of drugs from bilosomes. Third-factor, i.e. bile salt increases the EE (%) significantly (*p*<.0001) due to increased flexibility of bilosome, decreased the interfacial tension, and increased solubility of LL into the lipid bilayer (Chen et al., 2009; Guan et al., 2011). The fourth factor, i.e. PEG-2000 significantly (*p*<.0001) affects the EE (%) but has less impact than other factors. On increasing the PEG-2000 concentration, the EE (%) decreased due to the formation of pore into the membrane and hence leakage of the drug. A similar result showed by previous work (Albash et al., 2019). The ANOVA result showed that terms *A*, *B*, *C*, *D*, *AB*, *AC*, *AD*, *BD*, *A*^2^, *B*^2^, *C*^2^, *D*^2^ are significant (*p*<.05) model term, i.e. exhibited a positive impact on EE (%) and remaining terms exhibited non-significant (*p*>.5) effect on EE (Table 4). The model *F*-value was found to be 568.11 (*p*<.0001) which implies the model was well fitted with a non-significant (*p*>.05) lack of fit. The adequate precision <4 (89.739) designates an adequate signal for the fitted model. The predicted *R*^2^ of 0.9917 which is in reasonable agreement with the adjusted *R*^2^ of 0.9967 revealed that the quadratic model is well fitted.

### Selection of LL-PG-BLs-opt

Further, the optimized LL-PG-BLs (LL-PG-BLs-opt) were selected by the point prediction method of Box–Behnken’s design software. The composition of LL-PG-BLs-opt was found to be span 60 (45 mg), CHOR (25 mg), SDC (12.5 mg), and PEG-2000 (30 mg). The predicted values of VS and EE of LL-PG-BLs-opt were found to be 256 nm and 74%, respectively. The experimental values of VS and encapsulation efficiency were found to be 252 ± 3 nm and 75 ± 0.65, respectively. There was little variation between the actual and predicted value of responses which revealed that model was well fitted (Figure 3(A,B)). The desirability value of each factor was noted from the software. The value was found between 0.891 and 0.992, which confirms the used model was found suitable for optimization of NPs.

### Characterization

#### Vesicle characterization

**Figure 4.**
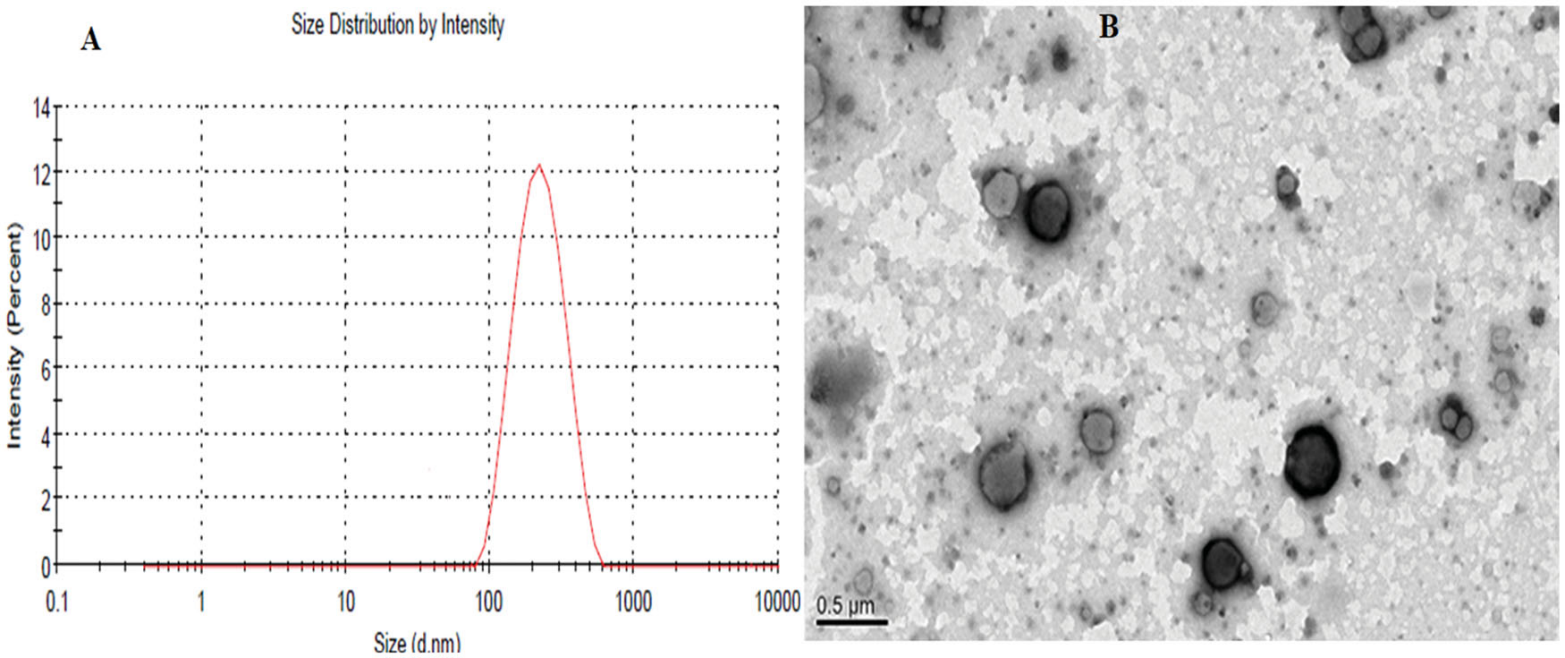
Showing the (A) particle size distribution graph and (B) TEM image of the optimized LL-PG-BLs-opt.

The LL-PG-BLs-opt exhibited VS 252 ± 3 nm **(**Figure 4(A)), PDI of 0.243 and ZP (–32 mV). The PDI value less than 0.05 indicates greater homogeneity of the particle size and the formulation will be stable for longer period of time. TEM image showed the vesicles were spherical without any visible aggregation (Figure 4(B)).

#### Thermal analysis

**Figure 5.**
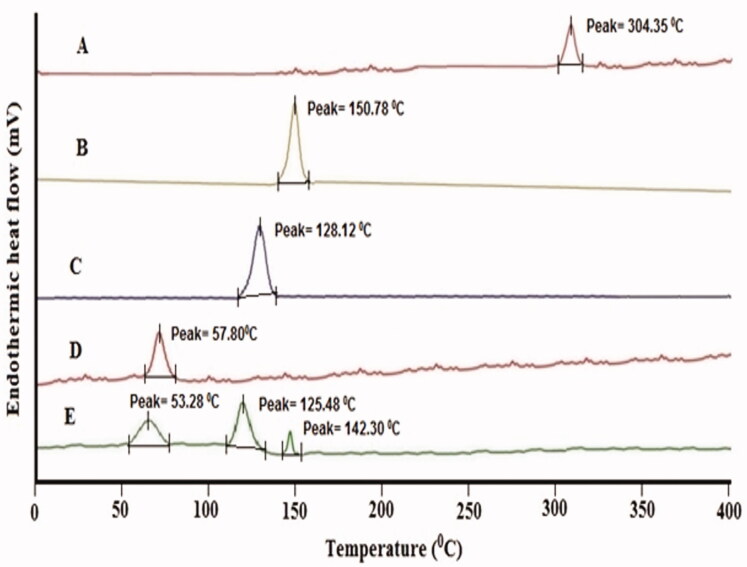
DSC curve: (A) pure luteolin, (B) bile salt, (C) polyethylene glycol 2000, (D) cholesterol, and (E) LL-PG-BLs-opt.

The thermal study was done on LL, BS, PEG2000, CHOR, and LL-PG-BLs-opt to evaluate the change in crystallinity of LL after loading into bilosomes **(**Figure 5). The thermogram of LL exhibited a high-intensity endothermic peak at 304.35 °C, equivalent to its melting point (Figure 5(A)). The characteristic endothermic peaks of BS, PEG2000, and CHOR, corresponding to their melting point were observed at 128.12 °C, 57.80 °C, and 150.78 °C, respectively (Figure 5(B–D)) (Albash et al., 2019). In LL-PG-BLs-opt thermogram, the characteristic peak of LL was completely disappeared while the peaks of BS and PEG2000 were slightly shifted to lower melting points 125.48 °C and 53.28 °C, respectively (Figure 5(E)) (Bhalekar et al., 2017). The reason for the absence of LL peak in the formulation suggested the complete entrapment of the drug in the lipids of the developed formulation. These results recommend a major interaction between drug and excipients of the bilayer structure which revealed the high stability of formulation (El-Sayed et al., 2017).

#### X-ray diffraction study

**Figure 6.**
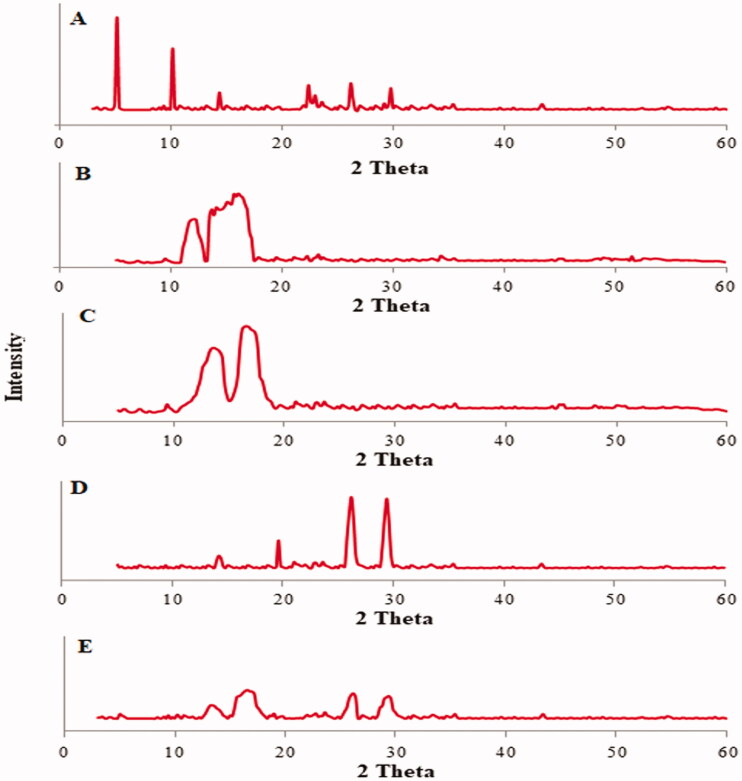
XRD-spectra: (A) pure luteolin, (B) cholesterol, (C) bile salt, (D) polyethylene glycol 2000, and (E) LL-PG-BLs-opt.

Figure 6 shows XRD spectra of LL, CHOR, BS, PEG-2000, and LL-PG-BLs-opt. The XRD spectrum of LL depicted the characteristics peaks with high-intensity at 5°, 10°, 22.4°, 23.5°, 26.2°, and 29.8° showing its crystalline nature (Figure 6(A)). CHOR exhibited intense peaks 14.4° (Figure 6(B)). However, BS showed the broad peaks at 13.6° and 16.6° (Figure 6(C)), and PEG-2000 had distinct sharp peaks at 14.2°, 19°, 26.2°, and 29.4° (Figure 6(D)). The spectrum of LL-PG-BLs-opt did not exhibit any characteristic peaks of LL. Some board peaks observed at 13.2°, 16.2°, 26°, and 29.2° in LL-PG-BLs-opt were corresponding to BS and PEG 2000 peaks (Figure 6(E)). The spectrum of optimized formulation revealed that the LL encapsulated into PG-BL matrix.

#### In vitro release study

**Figure 7.**
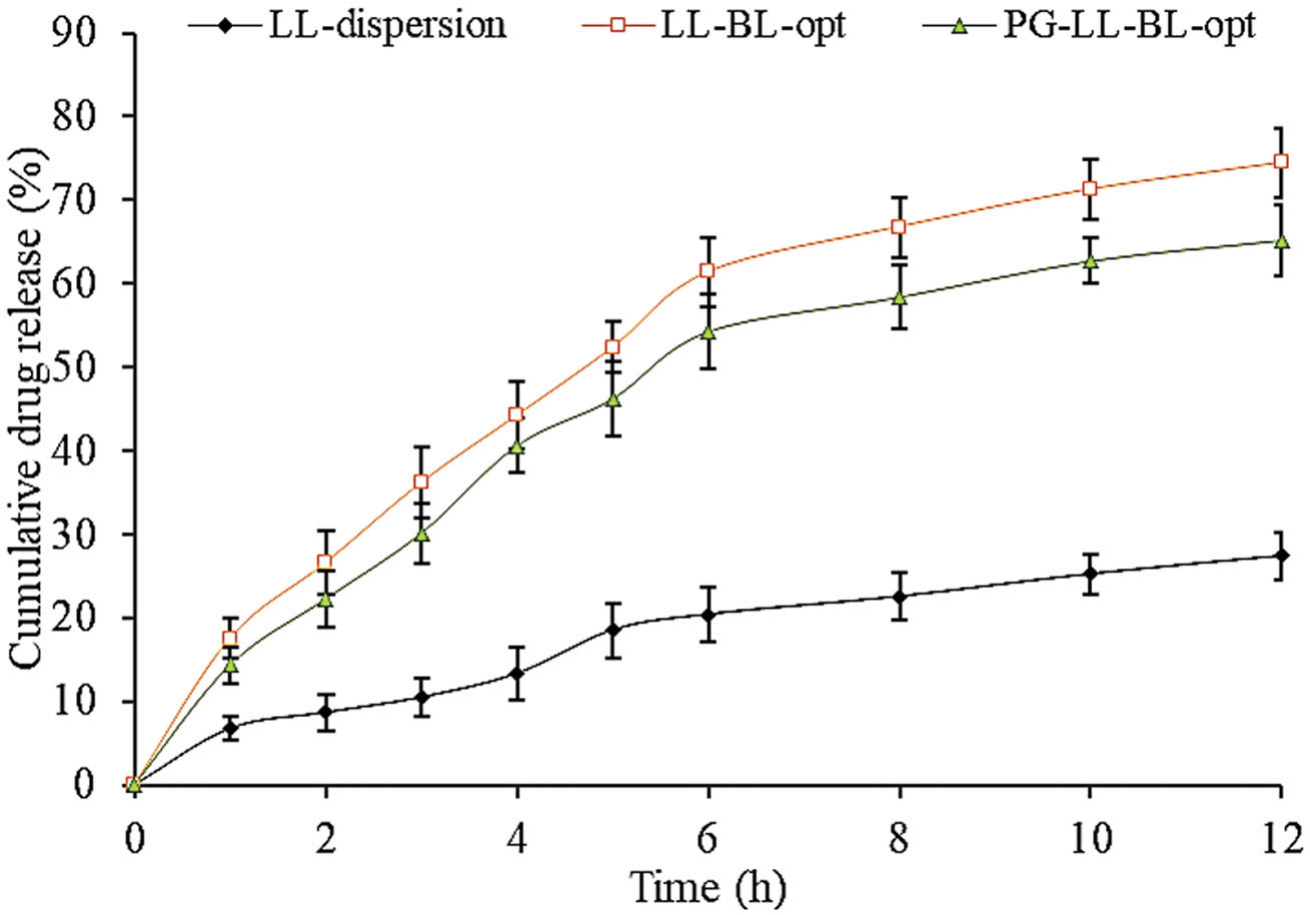
*In vitro* release profile of LL-dispersion, LL-BLs, and LL-PG-BLs-opt. Value represented as mean *±* SD (*n*= 3).

Figure 7 shows the comparative *in vitro* release profile of pure LL, LL-BLs-opt, and LL-PG-BLs-opt. LL-dispersion, LL-BLs-opt, and LL-PG-BLs-opt showed the release of 27.39 ± 1.55%, 74.45 ± 3.11%, and 65.24 ± 3.54%, respectively. LL-BL-opt and LL-PG-BLs-opt exhibited biphasic release with an initial fast release and later sustained release. The initial burst release might be due to the release of the drug from the surface of the vesicle. The slow release of LL from BLs was found due to the presence of CHOR which expectedly decrease the membrane fluidity (Nounou et al., 2006). The LL-BLs-opt and LL-PG-BLs-opt exhibited significantly (*p*<.05) higher LL release as compared to LL-dispersion. The higher release is due to the presence of surfactant and bile salt which increases the solubility of LL by reducing the interfacial tension. The LL-PG-BLs-opt formulation showed a significant (*p*<.05) sustained profile (65.24%) than LL-BL-opt (74.45%) due to pegylation (PEG-2000). The release profile of LL-PG-BLs-opt was fitted into kinetic models. The *R*^2^ value of all models was determined and found to be 0.8906 for first order, 0.9459 for first order, 0.9621 for Higuchi, 0.9774 for Korsmeyer-Peppas model, and 0.9477 for Hixson-Crowell model. The Korsmeyer-Peppas model was considered as the best fit model (*R*^2^=0.9774). The release exponents value (*n*) of 0.433 (<0.5) indicated the Fickian diffusion mechanism of drug release. The release profile of PG-BLs proposed that the LL would be stable in systemic circulation and slowly released at the site.

#### Permeation study

The permeation study of pure LL-dispersion, LL-BLs-opt, and LL-PG-BLs-opt was done by using excised rat gut. The amount of LL permeated was found to be 92.23 ± 3.87 µg for LL-dispersion, 714.54 ± 5.32 µg for LL-BLs-opt, and 1156.34 ± 4.67 µg for LL-PG-BLs-opt in 4 h, respectively. The flux of LL-BL-opt and LL-PG-BLs-opt was found to be 5.79- and 9.38-fold higher than LL-dispersion. The APC of LL-PG-BLs-opt exhibited significantly (*p*<.05) higher (5.1 × 10^−2^) than LL-BL-opt (3.17 × 10^−2^) and LL-dispersion (5.47 × 10^−3^). The LL-PG-BLs-opt showed significantly (*p*<.05) high permeation than LL-BLs-opt. It was suggested that PG-BLs were capable to permeate through the mucus barrier without any restriction whereas bilosomes may be trapped by the mucus barrier. Hence, it could be concluded that pegylation of bilosomes vesicle was permeated easily through the mucus of the intestine by the opening of the mucin layer (Yang et al., 2019).

#### In vitro antioxidant activity

**Figure 8.**
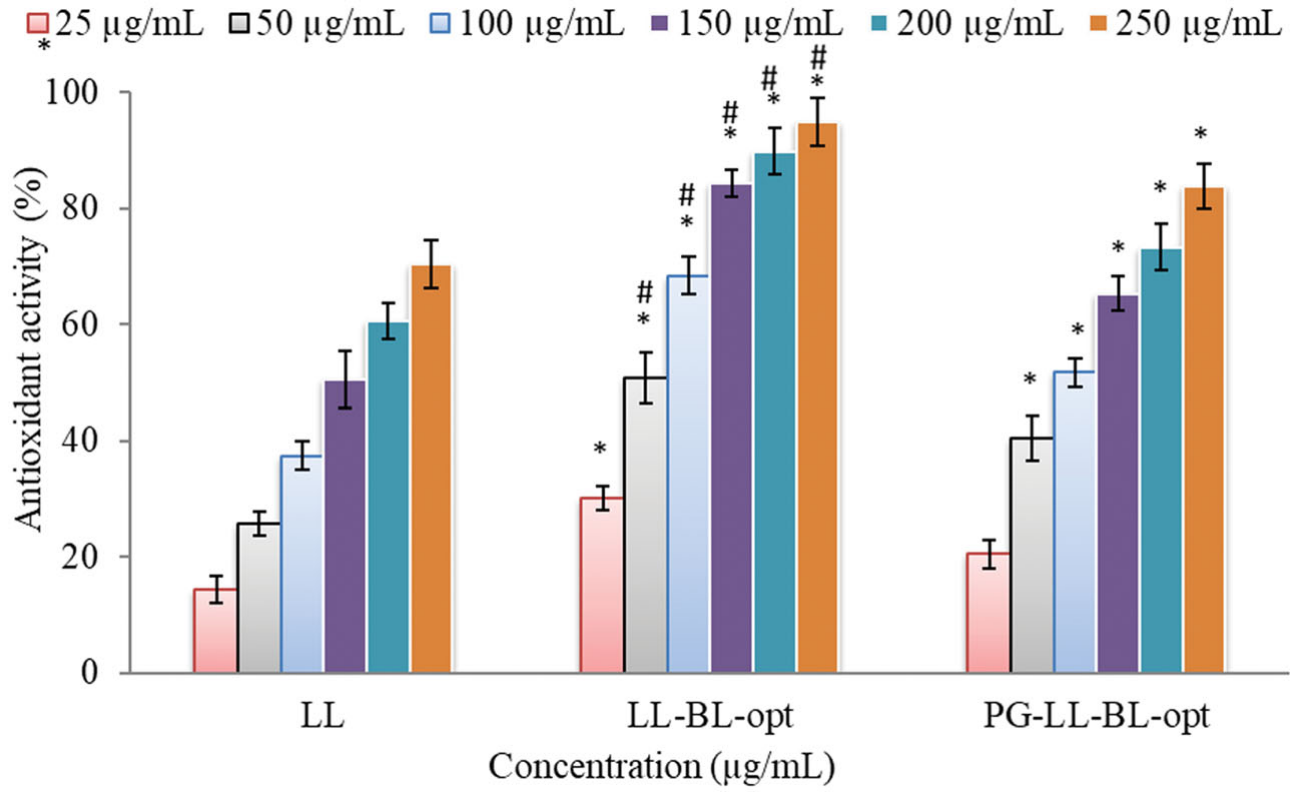
*In vitro* antioxidant activity of LL-dispersion, LL-BL-opt, and PG-LL-BL-opt. Data are the mean of three independent experiments and presented as mean ± SD, *significantly different from LL-dispersion (*p*<.05), ^#^significantly different from LL-BL-opt (*p*<.05).

The result of *in vitro* antioxidant activity of LL-dispersion, LL-BL-opt, and LL-PG-BLs-opt is depicted in Figure 8. It showed that the antioxidant potential of LL depends upon the concentration. On increasing the LL concentration, the antioxidant activity significantly (*p*<.05) increases. The order of antioxidant activity at different concentrations is LL-PG-BLs-opt > LL-BL-opt > pure LL. The maximum activity of pure LL-dispersion, LL-BLs-opt, and LL-PG-BLs-opt was found to be 70.31 ± 2.11%, 83.76 ± 2.65%, and 96.87 ± 3.32%, respectively, at 250 µg/mL concentration. The LL-PG-BLs-opt exhibited significant (*p*<.05) higher activity than LL dispersion and LL-BLs-opt. The higher activity of LL is achieved from LL-PG-BLs-opt due to the high solubility of LL in the pegylated liposome.

#### Cell viability study

**Figure 9.**
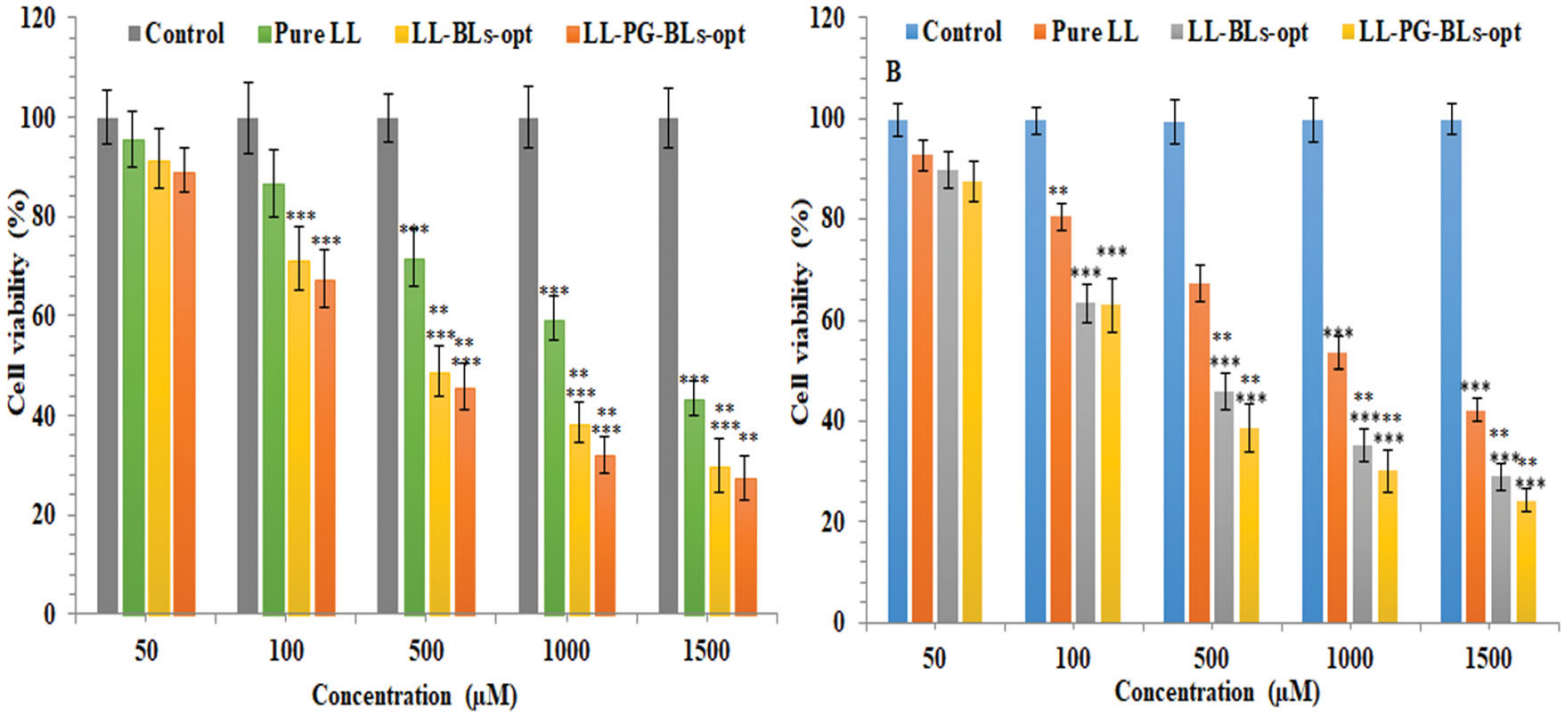
Cytotoxicity of LL incorporated in various formulations using MDA-MB-231 cell line at 24 h (A) and 48 h (B). Data shown are mean of three experiments and presented as mean ± SD. Tukey–Kramer’s multiple comparison test was used to evaluate the statistically significant difference between exposed different concentration and control. Difference was considered significant if *p*<.05. ***p<.001 when compared with control; **p<.001 when compared with the same concentration groups of pure LL.

**Figure 10.**
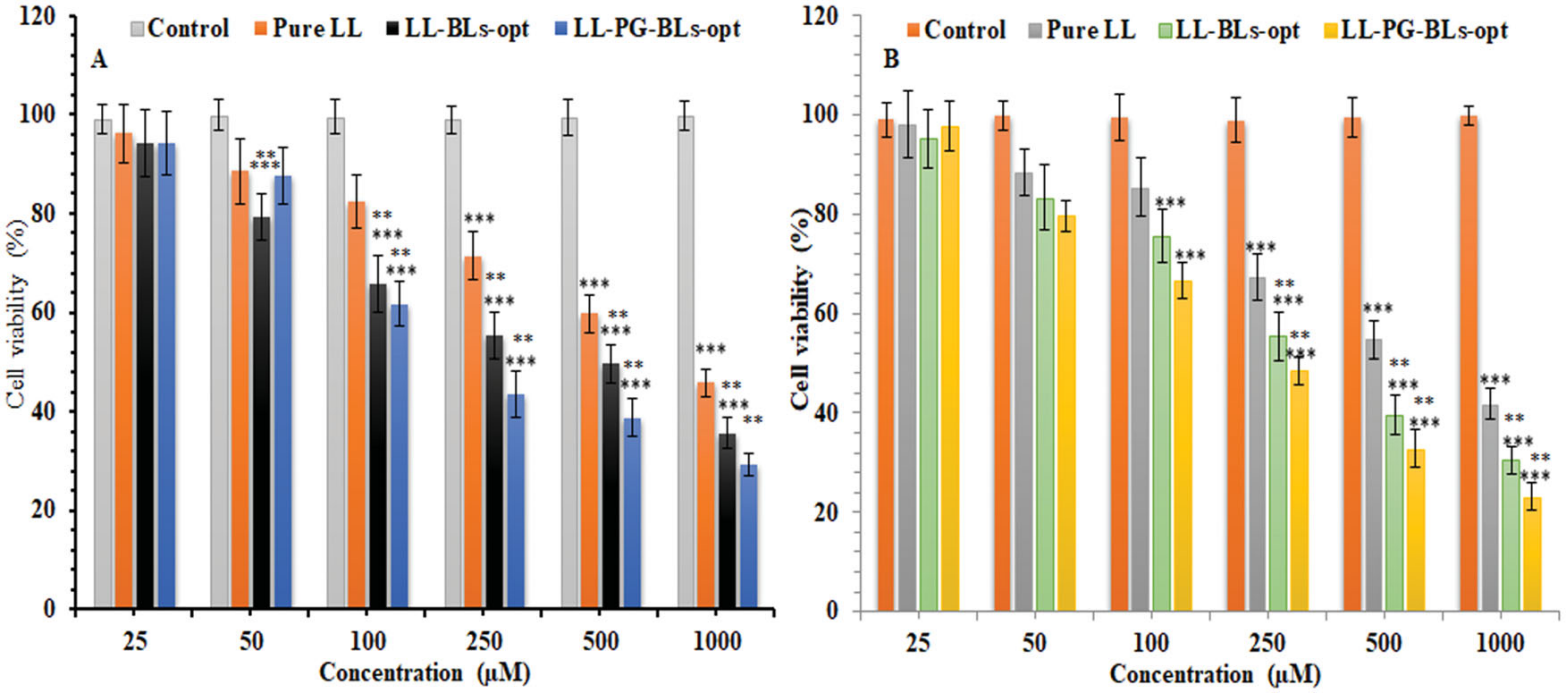
Cytotoxicity of LL incorporated in various formulation using MCF7 cell line at 24 h (A) and 48 h (B). Data shown are mean of three experiments and presented as mean ± SD. Tukey–Kramer’s multiple comparison test was used to evaluate the statistically significant difference between exposed different concentration and control. Difference was considered significant if *p*<.05. ***p<.001 when compared with control; **p<.001 when compared with the same concentration groups of pure LL.

Cell viability study of the prepared formulations was done on BC cell line, i.e. MDA-MB-231and MCF7. Figures 9 and 10 represent the % cell viability after the treatment with pure LL, LL-BL-opt, and LL-PG-BLs-opt at different concentrations in 24 h and 48 h. IC_50_ of pure LL, LL-BLs-opt, and LL-PG-BLs-opt in 24 h was found to be 1050 µM, 740 µM, and 610 µM, respectively, and at 48 h was found to be 980 µM, 650 µM, and 510 µM, against MDA-MB-231 cancer cell (Figure 9(A,B)). The order of cell inhibition on both cell lines is LL-PG-BLs-opt > LL-BL-opt > LL at tested concentration in 24 h and 48 h. The IC_50_ for pure LL, LL-BLs-opt, and LL-PG-BLs-opt in 24 h was found to be 920 µM, 480 µM, and 390 µM, respectively. At 48 h, IC_50_ value was found to be 820 µM, 410 µM, and 370 µM against MCF7 cancer cell (Figure 10(A,B)). It clearly showed that LL-PG-BLs-opt exhibited significantly high cell viability (***p<.05) on cell lines than LL-BLs-opt > LL. These high activities might be due to the higher release of LL from LL-PG-BLs-opt as well as high solubility of LL in pegylated formulation than LL-BLs-opt and pure LL (Esfahani et al., 2014; Ghaferi et al., 2020).

#### Antimicrobial activity

The antimicrobial study of pure LL, LL-PG-BLs-opt and pure ciprofloxacin solution was performed against *S. aureu*s and *E. coli.* The ZOI of pure LL, LL-PG-BLs-opt, and pure ciprofloxacin solution against *S. aureu*s was found to be 8.5 ± 0.14 mm, 16.25 ± 0.13 mm, and 17.85 ± 0.15 mm, respectively. However, ZOI of LL and LL-PG-BLs-opt against *E. coli* (gram-negative) was found to be 7.23 ± 0.11 mm, 14.67 ± 0.14 mm, and 18.45 ± 0.11, respectively. It was observed that LL-PG-BLs-opt exhibited significantly high (*p*<.05) antibacterial activity than pure LL and closed to pure ciprofloxacin solution. The significant-high activity of LL in the pegylated liposome is due to high stability, sustained release, and high solubility. The antibacterial activity of LL due to prevention of the nucleic acid and protein synthesis weakens cell membrane, as well as impedes the development of biofilm (Qian et al., 2020, 2021).

#### Stability study

**Figure 11.**
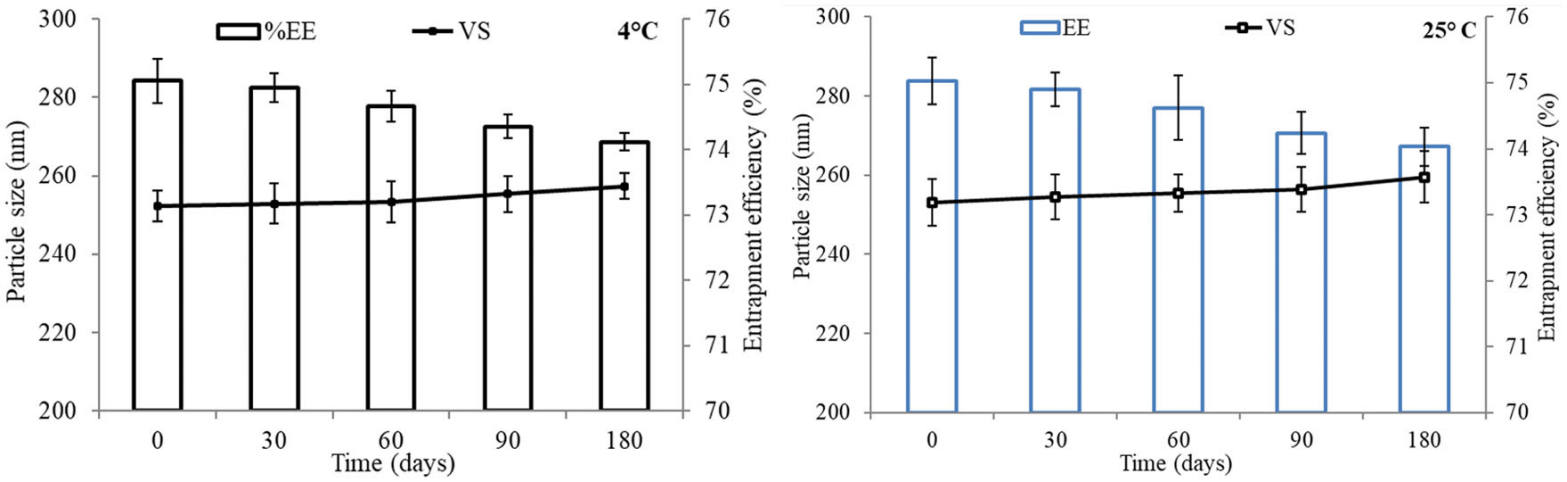
Stability study of optimized formulation (LL-PG-BL-opt) conducted at 4 °C and 25 °C. Experiment performed in triplicate and data shown as mean of three experiments (mean ± SD).

The stability study of LL-PG-BLs-opt was analyzed at 4 °C and 25 °C and results are depicted in Figure 11. No significant (*p*>.05) changes were observed in VS and EE. This may be due to the high negative charge owned by the presence of bile salt and PEG-2000 that prevents the grouping of the vesicles. In addition, due to low HLB value and high transition temperature, the vesicles were closely packed, preventing the leakage of the drug.

## Conclusions

In this study, PG-BLs were developed by the thin-film hydration method and optimized by four factors and three levels using the Box–Behnken design. The optimal formulation showed high EE, nano spherical vesicle with high LL release as well as flux. The LL-PG-BLs-opt exhibited significantly (*p*<.05) higher antioxidant activity than LL and LL-BL-opt dispersion. The formulation exhibits a promising anticancer activity against BC cell lines (MCF7 and MDA-MB-231). It was observed that LL-PG-BLs-opt exhibited significantly high (*p*<.05) antibacterial activity than pure LL against *S. aureus* and *E. coli*. Hence, the developed PG-BLs could be a promising delivery system for the delivery of LL for the management of BC with acceptable antioxidant and antimicrobial activities.
